# Experimental data on the production and characterization of biochars derived from coconut-shell wastes obtained from the Colombian Pacific Coast at low temperature pyrolysis

**DOI:** 10.1016/j.dib.2019.104855

**Published:** 2019-11-22

**Authors:** Deyler Castilla-Caballero, Juan Barraza-Burgos, Sundaram Gunasekaran, Aicardo Roa-Espinosa, José Colina-Márquez, Fiderman Machuca-Martínez, Aracely Hernández-Ramírez, Sofía Vázquez-Rodríguez

**Affiliations:** a Environmental Engineering and Chemical Engineering Programs, Universidad Tecnológica de Bolívar, Parque Industrial y Tecnológico Carlos Vélez Pombo km 1 Vía Turbaco, Cartagena, Colombia; b Escuela de Ingeniería Química, Universidad del Valle, Cali, A.A. 25360, Colombia; c Department of Biological Systems Engineering, University of Wisconsin, Madison, WI, 53706, USA; d Soil Net LLC, 560 Enterprise Ave, Belleville, WI, 53508, USA; e Chemical Engineering Program, Universidad de Cartagena, Av. El Consulado 48-152, Cartagena, A.A. 13001, Colombia; f Facultad de Ciencias Químicas, Universidad Autónoma de Nuevo León, Ave. Universidad S/N, Cd. Universitaria, San Nicolás de los Garza, N.L, C.P. 64450, Mexico; g Facultad de Ingeniería Mecánica y Eléctrica, Universidad Autónoma de Nuevo León, Pedro de Alba S/N, Cd. Universitaria, San Nicolás de los Garza, Nuevo León, 66455, Mexico

**Keywords:** Biochar, Coconut-shell, Low temperature pyrolysis, Oxygen variation, Characterization

## Abstract

Biochars are emerging eco-friendly products showing outstanding properties in areas such as carbon sequestration, soil amendment, bioremediation, biocomposites, and bioenergy. These interesting materials can be synthesized from a wide variety of waste-derived sources, including lignocellulosic biomass wastes, manure and sewage sludge. In this work, abundant data on biochars produced from coconut-shell wastes obtained from the Colombian Pacific Coast are presented. Biochar synthesis was performed varying the temperature (in the range: 280 °C–420 °C) and O_2_ feeding (in the range: 0–5% v/v) in the pyrolysis reaction. Production yields and some biochar properties such as particle size, Zeta Potential, elemental content (C, N, Al, B, Ca, Cu, Fe, K, Li, Mg, Mn, Na, P, S, Ti, Zn), BET surface area, FT-IR spectrum, XRD spectrum, and SEM morphology are presented. This data set is a comprehensive resource to gain a further understanding of biochars, and is a valuable tool for addressing the strategic exploitation of the multiple benefits they have.

**Table:** Specifications Table

Subject	Chemical Engineering::Chemical Engineering (General)
Specific subject area	Materials Science
Type of data	Figure and table
How data were acquired	Data were obtained by laser diffraction (Mastersizer 2000, Malvern Panalytical), phase-analysis light-scattering (90-Plus Particle-size and Zeta-Potential Analyzer, Brookhaven), combustion and reduction processes for total C and N content (Vario MAX cube, Elementar), inductively coupled plasma spectroscopy (TJA IRIS Advantage ICP/OES), BET analysis of nitrogen adsorption-desorption isotherms (Gemini VII, Micromeritics), IR spectroscopy (Tensor 27 spectrometer, Bruker), X-Ray diffraction spectroscopy (D8 Discover, Bruker), SEM microscopy (LEO 1530 SEM). Biochar yields were determined through mass recording in an analytical balance (0.0001 g).
Data format	Raw and analyzed
Parameters for data collection	The data were collected after varying pyrolysis temperature and oxygen content in the pyrolysis reaction used for producing biochars.
Description of data collection	Biomass samples for biochar synthesis were collected according to the ASTM C702/C702 M−18 method. Yields and characterization data were collected as indicated in the experimental section of this document.
Data source location	Biochar's synthesis was done in The Universidad del Valle, (Cali, Colombia) whereas its characterization was done in The Universidad Autónoma de Nuevo León (Monterrey, Mexico) and The University of Wisconsin (Madison, USA).
Data accessibility	Data is provided in this article.

**Table:** 

**Value of the Data** • The data describe valuable properties of biochars obtained from Colombian lignocellulosic wastes at low pyrolysis temperatures that may improve their further use and understanding. • Researchers in agricultural, environmental, material sciences, chemical, energy and related areas may benefit from the data presented in this work. • The data can be used to estimate biochar's production yields at larger scales. The chemical composition (elemental composition, FTIR) can be used to evaluate biochar effects on plant growing, plant productivity, nutrient fixation, soil amendment, water retention, and properties related thereon. Surface characterization (Zeta Potential, BET surface area, SEM) can be used to analyze adsorptive properties of the material. XRD, FTIR, and particle size measurements can be used to evaluate the compatibility of biochars as fillers in composite materials.

## 1 Data

**Fig. 1:**
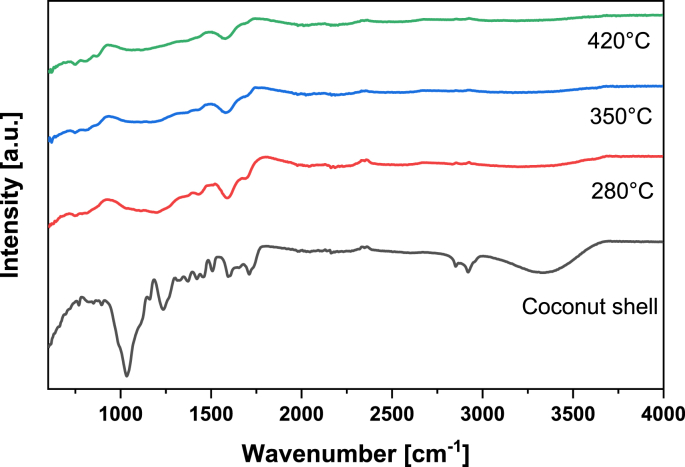
IR spectrum of biochars produced at different temperatures. The oxygen content in all cases was 2.5% v/v. The IR spectrum of the coconut shells is presented as a reference.

**Fig. 2:**
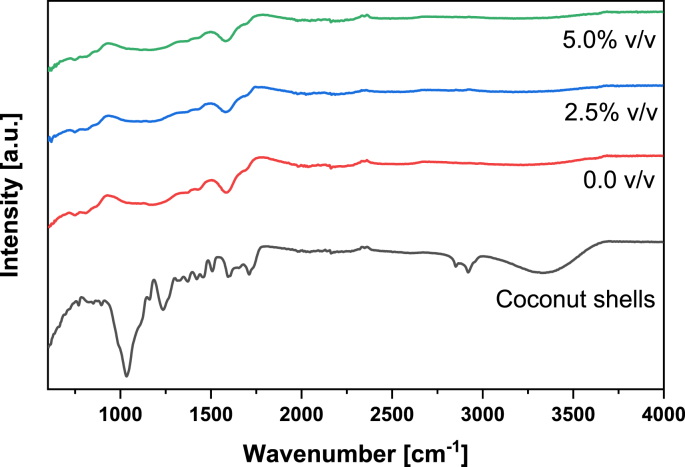
IR spectrum of biochars produced at different oxygen contents in the pyrolysis reactor. The temperature was 350 °C in all cases. The IR spectrum of the coconut shells is presented as a reference.

**Fig. 3:**
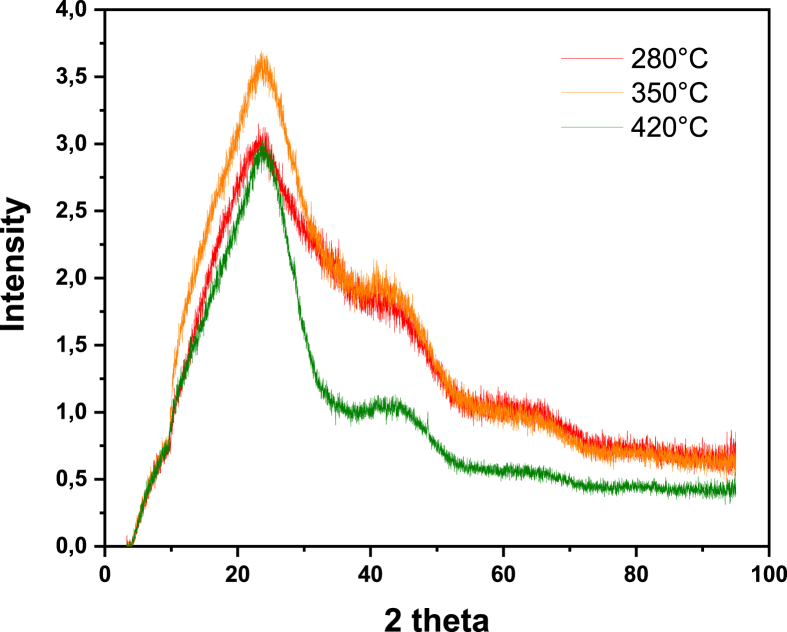
XRD spectrum of biochars produced at different temperatures. The oxygen content in the experiments was 2.5% v/v.

**Fig. 4:**
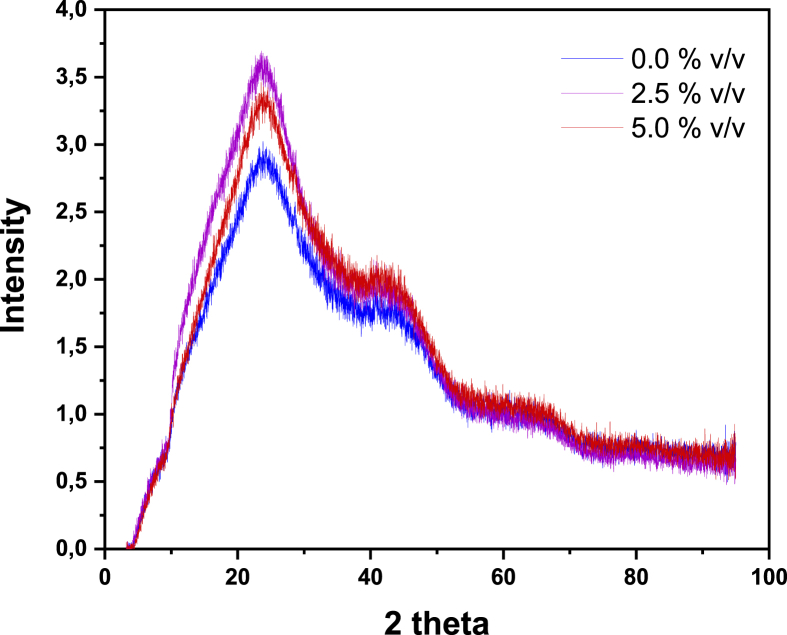
XRD spectrum of biochars produced at different values of oxygen content. The temperature in the experiments was 350 °C.

**Fig. 5:**
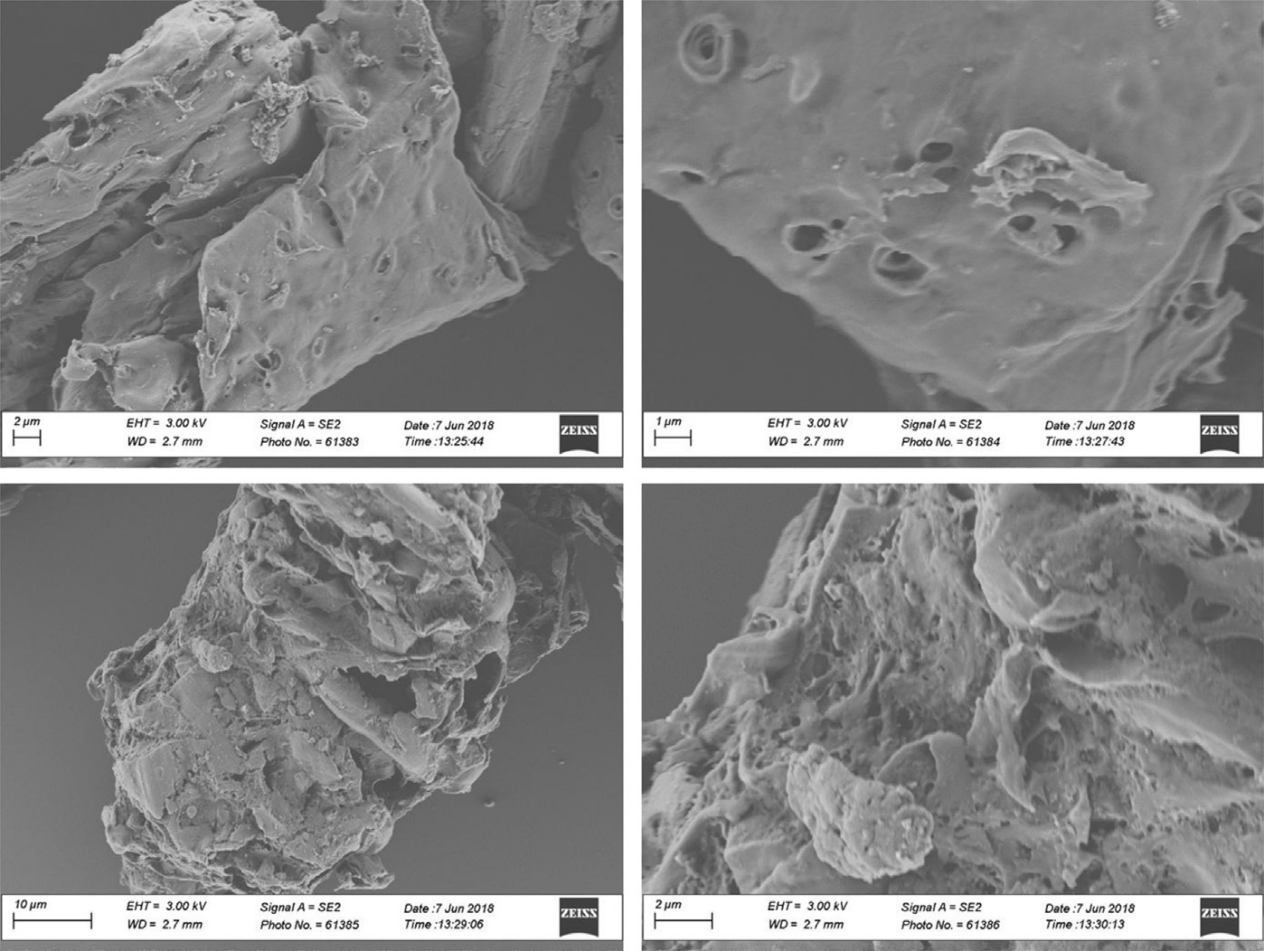
SEM morphology of biochar samples produced at 280 °C and 2.5% v/v of oxygen content.

**Fig. 6:**
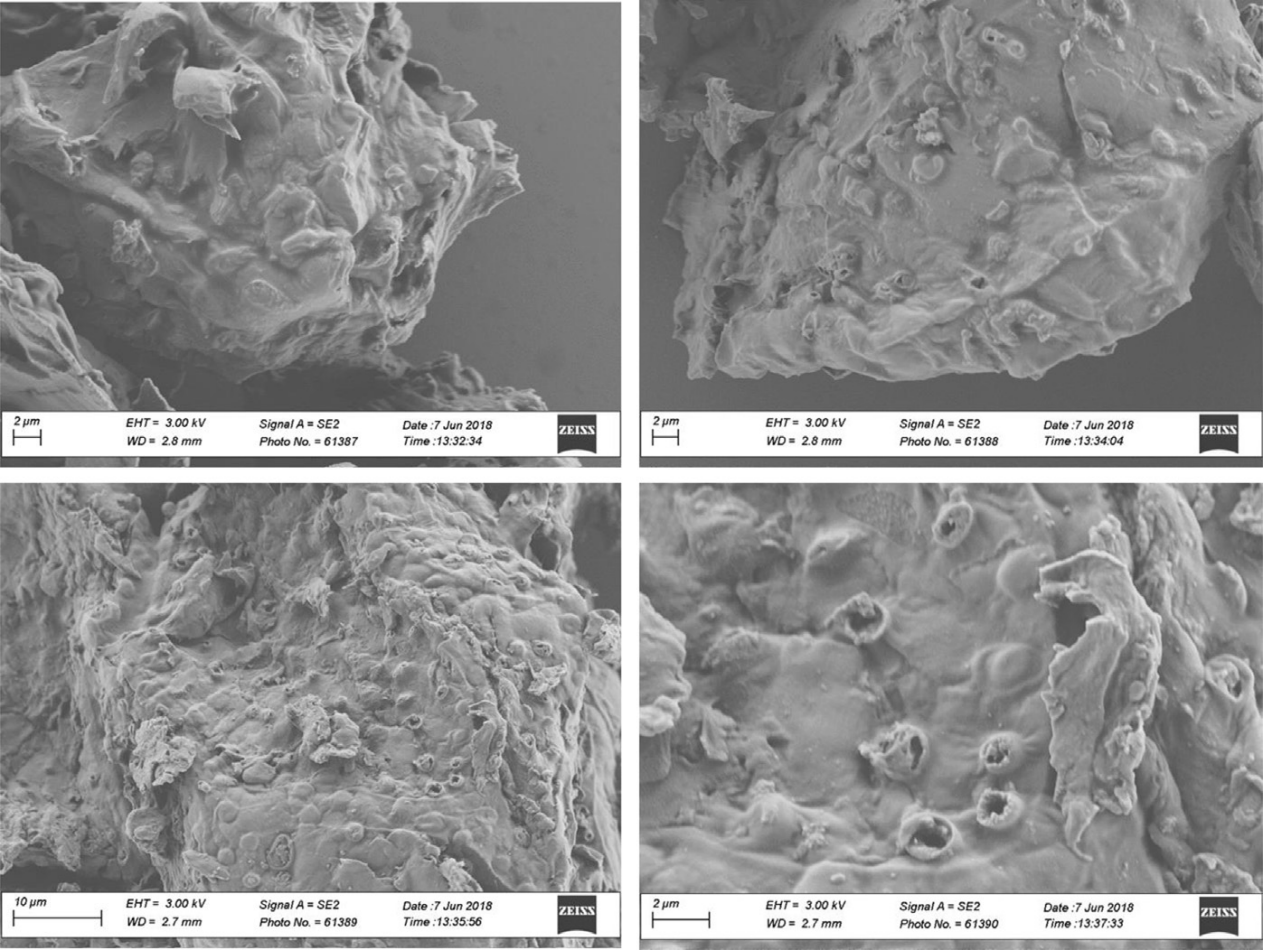
SEM morphology of biochar samples produced at 350 °C and 0% v/v of oxygen content.

**Fig. 7:**
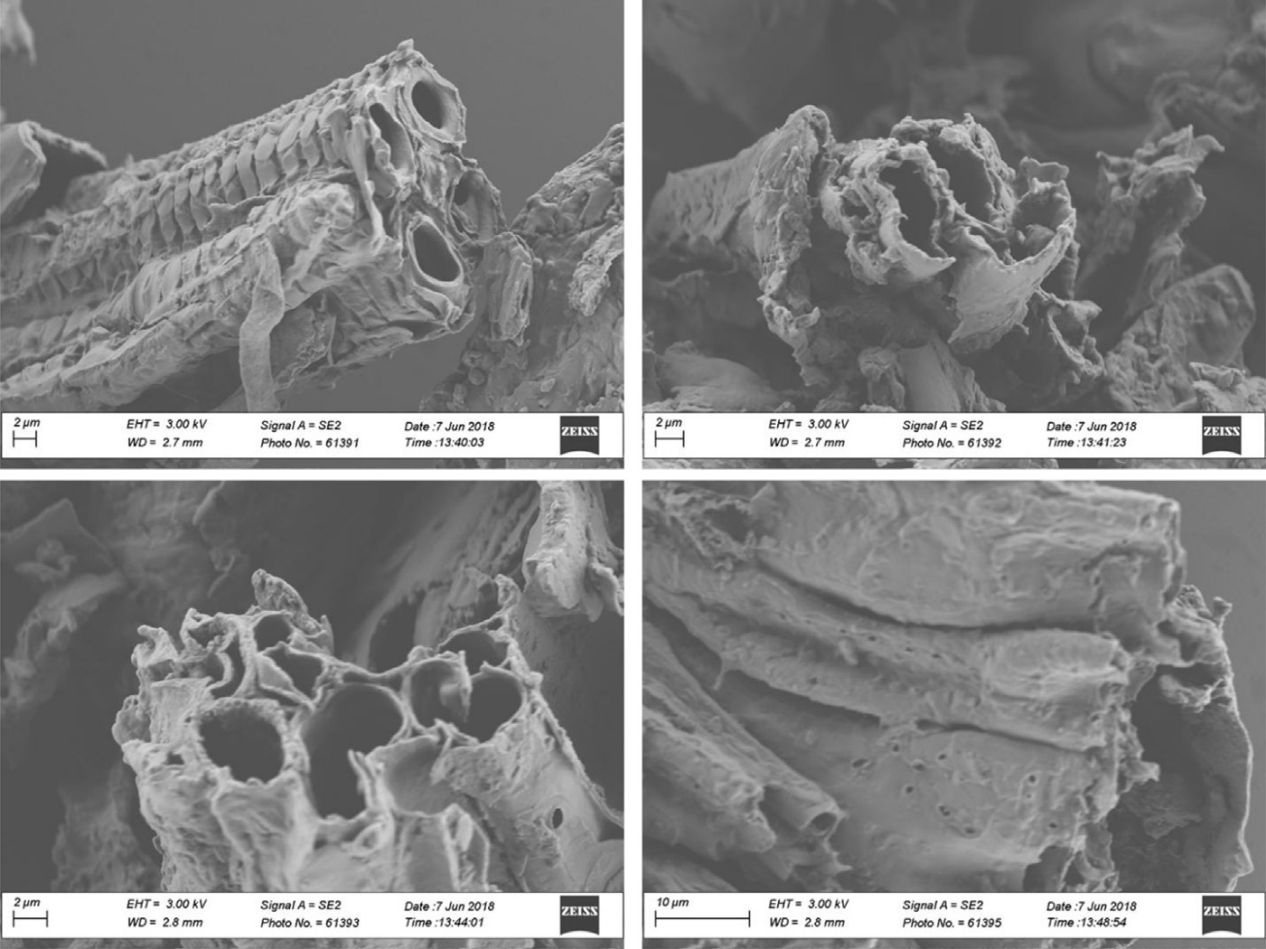
SEM morphology of biochar samples produced at 350 °C and 2.5% v/v of oxygen content.

**Fig. 8:**
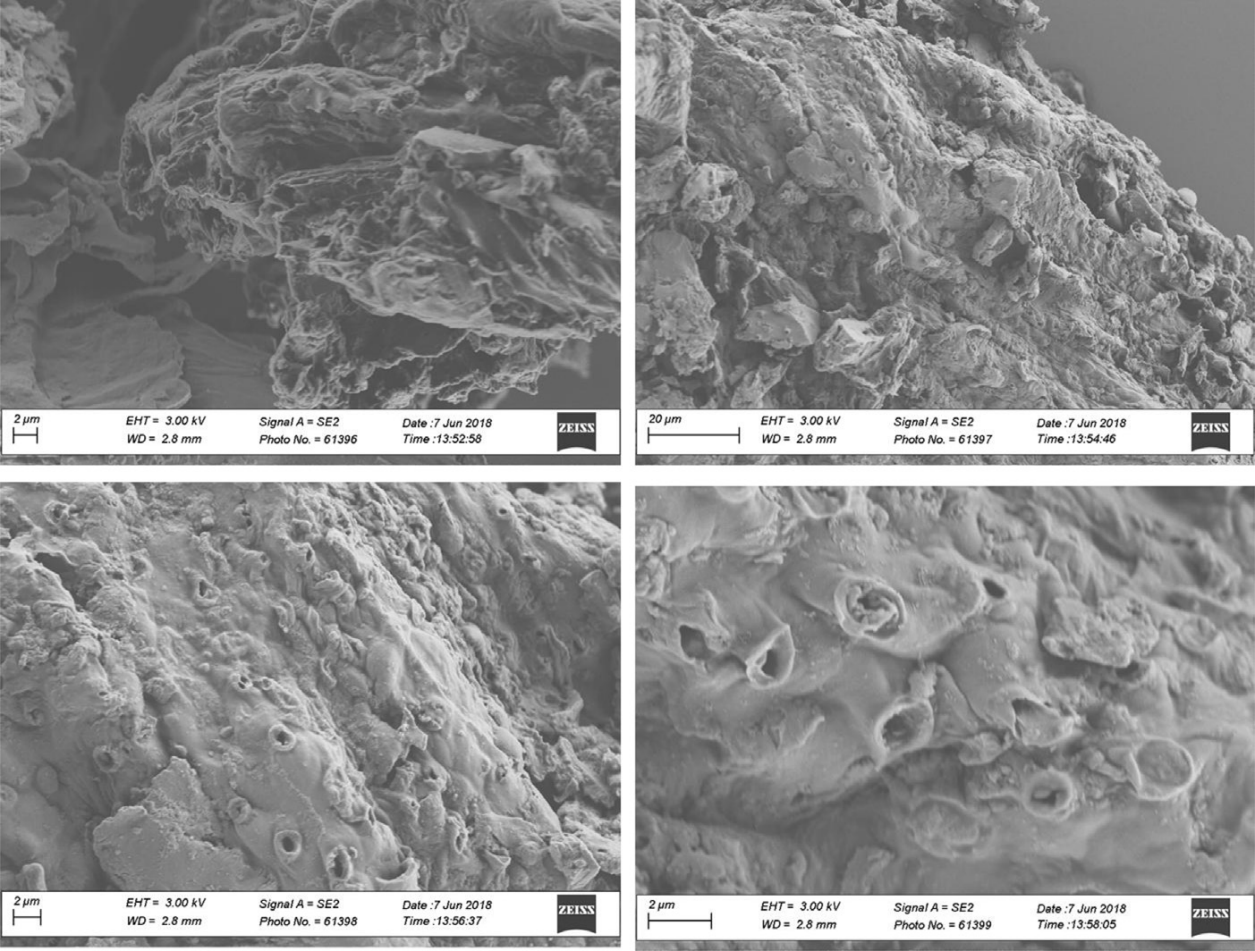
SEM morphology of biochar samples produced at 350 °C and 5% v/v of oxygen content.

**Fig. 9:**
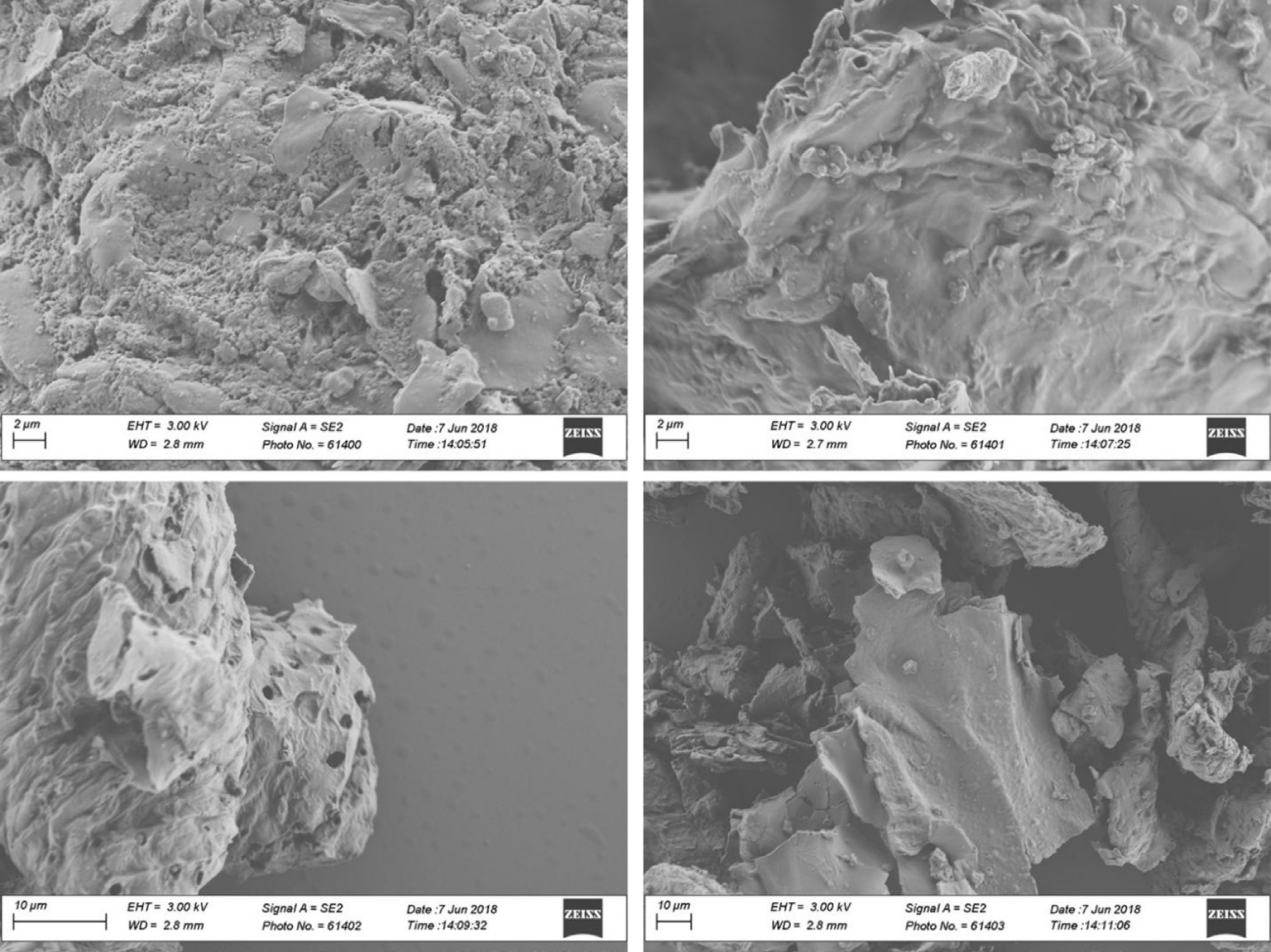
SEM morphology of biochar samples produced at 420 °C and 2.5% v/v of oxygen content.

The data presented in this work describe the production and characterization of biochars derived from coconut-shell wastes obtained from the Colombian Pacific Coast. The data correspond to the synthesis of biochar at different values of temperature and feeding of oxygen in the pyrolysis reaction. Table 1 presents the yields (%) of biochar, their mean particle size and Zeta Potential. Table 2 shows the total carbon and nitrogen contents of biochars. Table 3 displays the elemental content of biochars estimated through ICP spectroscopy. Table 4 shows the BET surface area of biochars. On the other hand, Fig. 1 and Fig. 2 plot the IR transmittance spectrum of biochars obtained at different values of temperature and feeding of oxygen in the pyrolysis reaction. In order to have a better understanding of these figures, the main transmittance IR-bands associated to the functional groups of the coconut shell and biochars are presented in Table 5. Besides, Fig. 3 and Fig. 4 show the XRD spectrum of biochars obtained at different values of temperature and feeding of oxygen in the pyrolysis reaction. In addition, Table 6 depicts the position of some reference XRD peaks for amorphous and crystalline carbonaceous materials, including biochars and graphite. Finally, Fig. 5, Fig. 6, Fig. 7, Fig. 8, Fig. 9 portray the morphology of the biochar samples through the SEM technique.

**Table 1:** Yields (%), mean particle size and Zeta Potential of biochars.

Pyrolysis temperature (°C)	Oxygen content (% v/v)	Yield of biochar (%)	Mean Particle size (μm)	Zeta Potential (mV)
280	2.5	36.17	119.33	−44.17
304	0.85	33.00	116.87	−26.79
304	4.14	32.66	114.93	−37.04
350	0	32.33	115.69	−41.76
350	2.5	30.43	114.24	−45.41
350	5	29.86	114.32	−45.05
396	0.85	28.62	113.10	−36.74
396	4.14	28.14	108.34	−36.10
420	2.5	27.13	113.73	−41.15

**Table 2:** Total Carbon and Nitrogen contents of biochars.

Pyrolysis temperature (°C)	Oxygen content (% v/v)	Total Carbon content (% wt.)	Total Nitrogen content (% wt)
280	2.5	53.35	0.49
304	0.85	45.60	0.45
304	4.14	39.93	0.44
350	0	50.78	0.46
350	2.5	36.70	0.41
350	5	46.31	0.45
396	0.85	39.64	0.40
396	4.14	42.47	0.42
420	2.5	43.42	0.41

**Table 3:** Elemental analysis of coconut shells and biochars through ICP spectroscopy.

Pyrolysis temperature (°C)	Oxygen content (% v/v)	Al (ppm)	B (ppm)	Ca (ppm)	Cu (ppm)	Fe (ppm)	K (ppm)	Li (ppm)
Coconut Shell		26.64	9.60	177.23	12.78	1541.62	2752.84	0.50
280	2.5	–	14.23	421.24	30.98	3755.75	7712.83	1.02
304	0.85	182.21	548.46	612.94	26.19	3756.40	9517.20	1.13
304	4.14	115.54	245.85	502.74	29.03	4271.60	8650.97	1.19
350	0	216.91	579.31	640.02	31.18	4280.24	8973.81	1.23
350	2.5	133.49	12.78	428.90	31.51	3651.71	6694.86	1.76
350	5	50.85	16.79	486.39	27.30	4340.98	8999.59	1.13
396	0.85	249.55	540.14	725.91	27.70	4475.68	9987.73	1.30
396	4.14	669.05	934.47	829.87	37.50	4954.65	11534.96	1.49
420	2.5	66.90	20.69	546.91	33.84	4966.18	9264.71	1.51

Pyrolysis temperature (°C)	Oxygen content (% v/v)	Mg (ppm)	Mn (ppm)	Na (ppm)	P (ppm)	S (ppm)	Ti (ppm)	Zn (ppm)
Coconut shell		86.30	13.40	393.38	406.52	354.72	0.90	16.48
280	2.5	473.23	33.96	1079.20	1034.29	451.31	10.60	25.99
304	0.85	460.60	32.62	2878.80	1048.28	508.26	8.42	0.00
304	4.14	360.68	40.53	1745.00	1066.17	459.47	9.68	28.40
350	0	470.48	37.14	2392.86	1144.67	449.50	11.76	27.27
350	2.5	544.86	35.63	1045.65	2550.24	401.03	13.46	25.46
350	5	371.52	43.13	1267.07	1155.94	442.25	12.36	30.61
396	0.85	523.41	38.65	2614.55	1178.48	446.09	10.39	29.63
396	4.14	545.58	43.53	3744.47	1336.84	512.70	9.80	32.36
420	2.5	389.12	41.47	1305.68	1266.00	498.56	10.24	41.46

**Table 4:** BET surface area (m^2^/g) of biochars.

Pyrolysis temperature (°C)	Oxygen content (% v/v)	BET surface area (m^2^/g)
280	2.5	13.28125
304	0.85	10.9311
350	2.5	15.5675
396	4.14	15.7544
420	2.5	9.8468

**Table 5:** Main IR-transmittance bands for the functional groups of the coconut shell and biochars. Adapted from Refs. [1,2].

Wavenumber [cm^−1^]	Characteristic vibrations	Functionality
3200–3500	O–H stretching	Water, H-bonded hydroxyl (-OH) groups
∼ 2935	Asymmetric C–H stretching	Aliphatic CH_x_
∼ 2885	Symmetric C–H stretching	Aliphatic CH_x_
1700–1740	C=O stretching	Mainly Carboxyl, traces of aldehydes, ketones and esters
∼ 1600	C=C stretching together with C=O stretching	Aromatic compounds
∼ 1030	Symmetric stretching of C–O–C	Aryl-alkyl ethers, functional groups of cellulose, hemicellulose and lignin
750–885	C–H bending	Aromatic C–H out-of-plane deformation

**Table 6:** Some reference XRD peaks for amorphous and crystalline carbonaceous materials. Taken from Refs. [3,4].

2 Theta (Bragg) Angle	Crystallographic form
Broad peak at ∼24	Graphite-like structure (turbostratic carbon, amorphous)
Broad peak at ∼42	Graphite-like structure (turbostratic carbon, amorphous)
Sharp intense peaks at ∼ 26, ∼ 44, and ∼ 55, and less sharp peak at ∼60	Graphite (crystalline)

## 2 Experimental design, materials, and methods

**Fig. 10:**
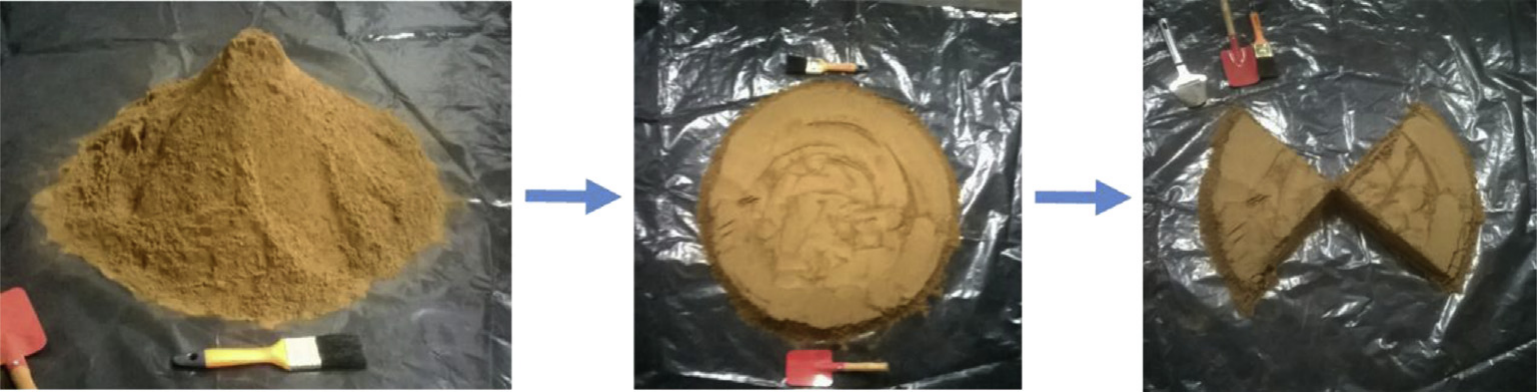
Sampling (ASTM C702/C702 M−18) of grinded coconut shells used for the synthesis of biochar.

**Fig. 11:**
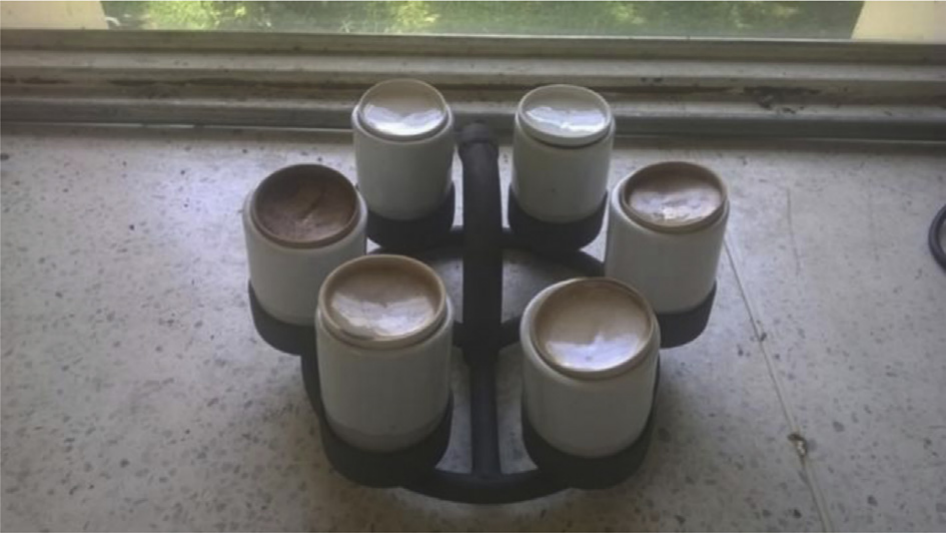
Pyrolysis reactors.

**Synthesis of biochars**: Coconut shell wastes from the Colombian Pacific Coast were used as biochar precursors. These shells were grinded using a jaw crusher and a hammer mill to get enough amounts of particles less than 250 μm. Next, sampling was done according to the quartering method described in the ASTM C702/C702 M standard [5] (See Fig. 10) as follows: the original sample was placed in a clean, level plastic surface. Then, the material was mixed thoroughly by turning the entire sample several times. After the last turning, the entire sample was shoveled into a conical pile by depositing each shovelful on top of the preceding one. The pile was then flattened by pressing the top without further mixing, and afterwards, it was divided into four equal quarters by cutting two diameters at right angles. Finally, two diagonally opposite quarters were removed, and the remaining quarters were mixed and taken to the next stage of the process, in which the grinded particles were fed into the system of six parallel closed fixed-bed-pyrolysis reactors shown in Fig. 11.

**Fig. 12:**
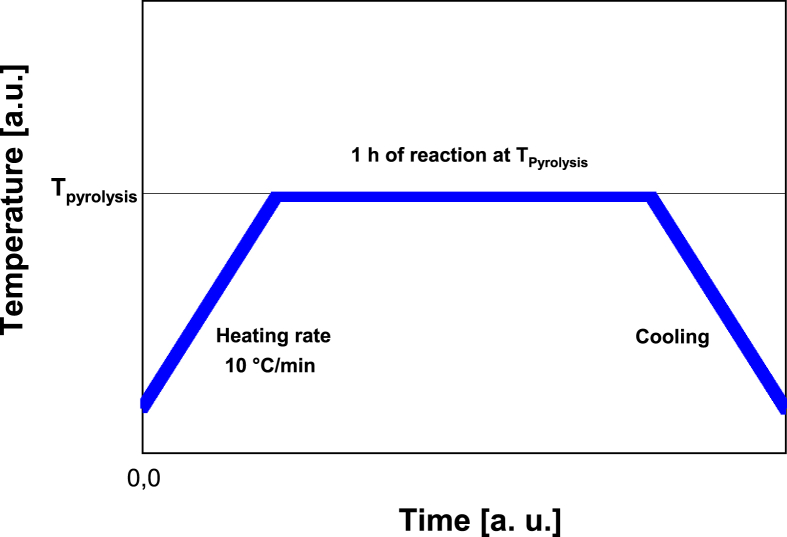
Temperature profile scheme during pyrolysis experiments.

During the pyrolysis experiments, the temperature and O_2_ content were modified according to the experimental design shown in Table 7. In a typical experiment, each of the 6-pyrolysis reactors was loaded with ∼25 g of grinded coconut shells. Then, they were placed inside a muffle furnace that was previously set at the desired temperature. After that, the N_2_ and O_2_ feeding were allowed to flow into the muffle at a specific rate to maintain the O_2_% v/v value according to the experimental design. In all cases, the total gas flowrate (N_2_ plus O_2_) into the muffle was set at 4210 ml/min, the heating rate was taken as 10 °C/min, and the reaction time at the desired temperature was 1 hour (See scheme in Fig. 12). Once the heating time at the desired temperature was reached, the N_2_ and O_2_ gases were allowed to keep flowing into the system until the reactor's temperature dropped to 100 °C. Then, the reactors were taken out from the furnace, and allowed to cool down through natural convection up to ∼30 °C. At that point, biochar was collected in resealable bags and was ready for further use.

**Table 7:** Experimental design for the synthesis of biochar.

Experiment	Pyrolysis temperature (°C)	Oxygen content (% v/v)
1	280	2.50
2	304	0.85
3	304	4.14
4	350	0.00
5	350	2.5
6	350	5.00
7	396	0.85
8	396	4.14
9	420	2.50

*Biochar yields* were estimated according to equation (1):

(1) $$\text{\% Biochar yield}=\frac{\text{Weight of produced biochar }}{\text{Weight of coconut shell fed to the reactors}}\times 100$$

### 2.1 Characterization of biochar

**Particle size distribution** of biochar samples were estimated through laser diffraction with a Mastersizer 2000 (Malvern Panalytical). The agitation rate was set at 2000 RPM and the refraction and absorption indexes were taken as 2.42 and 1.0 respectively.

**The total carbon and nitrogen content** of biochars were measured through combustion and reduction processes with a Vario MAX cube-elemental Analyzer in CN mode. In this case, ∼300 mg of each sample were initially loaded in the combustion chamber and sulfadiazine was used as the calibration reference standard.

**Surface charge** was estimated through Zeta Potential measurements in a 90-Plus Particle-size and Zeta-Potential Analyzer (Brookhaven). This instrument uses the phase-analysis light-scattering technique and required the samples to be pre-treated as follows: biochars were passed through a No. 200 sieve (<75 μm) and dried in a vacuum oven at 70 °C during 4 h. Then, 0.025 g of the sample were weighted in a clean 30-ml plastic tube and 25 ml of deionized water were added. The suspension was vortexed during 20 s after which an aliquot was transferred to the Zeta-Potential machine for its analysis. The pre-treatment step was adapted from Ref. [6]. The pH of the deionized water was ∼ 6.7.

**The elemental content** of biochars (Al, B, Ca, Cu, Fe, K, Li, Mg, Mn, Na, P, S, Ti, Zn) was measured through inductively coupled plasma (ICP) spectroscopy using a TJA IRIS Advantage ICP/OES (Sample uptake rate: 1.2 ml/min, acquisition time: 30 s in the low wavelength range and 10 s in the high wavelength range). In order to use of ICP spectroscopy, biochar samples were previously digested as follows: 0.1 g of the samples were weighted and placed in the digestion tubes. Then, 5 ml of HNO_3_ (trace metal grade) were added to the tubes which were subsequently heated at 120 °C for 12 hours inside a fume hood. Next, the system was let to cool down for 10 min after which 1 ml of hydrogen peroxide was added. The system was taken back to the fume hood to be heated again for 30 min. The last step was repeated, although in this case 2 ml of hydrogen peroxide were added. Finally, the digested samples were let to cool down and were diluted with deionized water for its subsequent use in the ICP apparatus.

**Surface area** of biochar was estimated through the BET (Brunauer-Emmett-Teller) theory, analyzing the N_2_ adsorption/desorption isotherms in a Gemini VII (Micromeritics). N_2_ isotherms were measured at −196 °C and the samples were previously degassed under vacuum at 150 °C during 2 h, as recommended in Ref. [7].

**The IR transmittance spectrum** of the biochar and coconut-shell samples was recorded in a Bruker TENSOR 27 (FT-IR) spectrometer in ATR mode. The samples were analyzed in the range of 600 and 4000 cm^−1^, performing 20 scans in each run, using a resolution of 4 cm^−1^.

**X-Ray diffraction** spectrums were recorded in a D8 DISCOVER Family diffractometer. Biochars powders were exposed to Cu Kα radiation with a wavelength (λ) 0f 1.5408 Å in the instrument. The incidence-angle (θ) range was chosen as 10–50° with a step time of 60 s/step and a step size of 10°/step.

**Biochars morphology** was monitored through SEM microscopy using a LEO 1530 SEM operated at 3 kV. Prior to measurements, diluted suspensions of biochar were prepared by dispersing biochar particles in deionized water under sonication. Afterwards, small aliquots of the suspensions were deposited on silicon wafers and were air-dried overnight. Next, the samples were gold sputtered during 50 s and then were ready for SEM scanning.
